# The Role of Dietary Fiber in the Bioaccessibility and Bioavailability of Fruit and Vegetable Antioxidants

**DOI:** 10.1111/j.1750-3841.2010.01957.x

**Published:** 2011-01

**Authors:** Hugo Palafox-Carlos, Jesús Fernando Ayala-Zavala, Gustavo A González-Aguilar

**Keywords:** antioxidants, bioaccessibility, bioavailability, dietary fiber, fruits and vegetables

## Abstract

Antioxidants are abundant compounds primarily found in fresh fruits and vegetables, and evidence for their role in the prevention of degenerative diseases is continuously emerging. However, the bioaccessibility and bioavailability of each compound differs greatly, and the most abundant antioxidants in ingested fruit are not necessarily those leading to the highest concentrations of active metabolites in target tissues. Fruit antioxidants are commonly mixed with different macromolecules such as carbohydrates, lipids, and proteins to form a food matrix. In fruits and vegetables, carbohydrates are the major compounds found, mainly in free and conjugated forms. Dietary fiber, the indigestible cell wall component of plant material, is considered to play an important role in human diet and health. Most studies on antioxidant bioavailability are focused on foods and beverages from which antioxidants are easily released. There is evidence indicating that food microstructure affects the bioaccessibility and bioavailability of several nutrients, referring mostly to antioxidants. Nevertheless, the specific role of dietary fiber in the absorption of antioxidants has not been widely discussed. In this context, the purpose of the present review is to compile and analyze evidence relating to the association between dietary fiber and antioxidants, and the physical and chemical interactions that modulate their release from the chyme in the gastrointestinal tract.

## Introduction

In recent years, the relationship between fruit and vegetable intake and health has been the focal point of much scientific investigation, in attempts to identify the specific plant components that convey health benefits. Fruits and vegetables, apart from being good sources of vitamins, minerals, and fiber, are also rich sources of potentially bioactive compounds known as phytochemicals. Antioxidants are abundant phytochemicals that prevent some of the processes involved in the development of cancer and cardiovascular disease (Denny and Buttriss 2007). Evidence for their role in the prevention of other diverse degenerative diseases is continuously emerging.

The bioaccessibility and bioavailability of each antioxidant differs greatly, and the most abundant antioxidants in ingested fruit are not necessarily those leading to the highest concentrations of active metabolites in target tissues (Manach and others 2005). Several factors interfere with the bioavailability of antioxidants, such as food source and chemical interactions with other phytochemicals and biomolecules present in the food (Parada and Aguilera 2007). Fruit antioxidants are commonly mixed with different macromolecules such as carbohydrates, lipids, and proteins to form the food matrix. In plant tissue, carbohydrates are the major compounds found, mainly in free and conjugated forms (Manach and others 2004).

Dietary fiber, the indigestible cell wall component of plant material, is considered to play an important role in human diet and health (Adiotomre and others 1990; Prosky 2000; Montagne and others 2003; Cummings and others 2004; DeVries 2004). However, there is evidence indicating that these complex carbohydrates directly interact with the food antioxidants and interfere with the adequate assimilation of these compounds (Faulks and Southon 2005; Parada and Aguilera 2007; Porrini and Riso 2008; Del Rio and others 2009; Pérez and others 2009). Most studies on antioxidant bioavailability are focused on foods and beverages from which antioxidants are easily released (Furr and Clark 1997; Ferguson and others 2004; Lafay and others 2006; Lafay and Gil-Izquierdo 2008). Research concerning the bioaccessibility of phenolic compounds and other antioxidants from solid matrices are important, since only the compounds released from the food matrix and/or absorbed in the small intestine are potentially bioavailable and in a condition to exert their beneficial effects (Tagliazucchi and others 2009).

Previous studies have reviewed the evidence indicating that the food microstructure affects the bioaccessibility and bioavailability of several nutrients, referring mostly to antioxidants (Parada and Aguilera 2007). Nevertheless, the specific role of dietary fiber in the absorption of antioxidants has not been widely discussed. In this context, the purpose of the present review is to compile and analyze evidence concerning the association of dietary fiber with antioxidants during ingestion of fruit and vegetables, through physical and chemical interactions that modulate their release from the chyme to the gastrointestinal tract.

### Link between fruit and vegetable intake and human health

The link between diet and health has been recognized, since ancient times when physicians treated their patients with herbs and foods believed to have medicinal properties (Steinmetz and Potter 1996). Several epidemiological studies have shown an association between the consumption of diets rich in fruits and vegetables and a lowered risk for chronic diseases such as cancer (Steinmetz and Potter 1996; Vainio and Weiderpass 2006), heart disease (Hertog and others 1993; Joshipura and others 2001), and stroke (Gillman and others 1995; Joshipura and others 1999). A reduced risk of obesity and better control of diabetes are some additional benefits that are likely to follow from increased consumption of plant foods (Steinmetz and Potter 1996). While the specific patterns of plant components responsible for this association remain to be established, there is evidence for a greater effect at higher levels of fruit and vegetable consumption, in line with current recommendations to increase fruit and vegetable intake to a minimum of 400 g/d (Nishida and others 2007; Del Rio and others 2009).

Fruits and vegetables, apart from being good sources of vitamins, minerals, and fiber, are also rich sources of potentially bioactive compounds known as phytochemicals. These compounds are not considered as nutrients, but much of the disease prevention potential of fruits and vegetables in human health is thought to be provided by these compounds (Denny and Buttriss 2007; Del Rio and others 2009; Gonzalez-Aguilar and others 2010).

A popular explanation among scientists, and more recently in the mass media, has been that food components with antioxidant properties (including vitamins C and E, selenium, flavonoids, and ß-carotene) present in these foods may prevent some of the processes involved in the development of cancer (for example, by protecting DNA from oxidative damage) and in the development of cardiovascular disease (for example, by inhibiting oxidative damage to LDL-cholesterol) (Denny and Buttriss 2007).

### Generalities of phenolic compounds and carotenoids

Several thousand known molecules having a phenolic structure (that is, several hydroxyl groups on aromatic rings) have been identified in higher plants, and several hundred are found in fruits and vegetables (Manach and others 2005; Denny and Buttriss 2007). These molecules are secondary metabolites of plants and are generally involved in defense against ultraviolet radiation or physiological damage by pathogens (Denny and Buttriss 2007). These compounds may be classified into different groups as a function of the number of phenol rings that they contain and of the structural elements that bind these rings to one another (Manach and others 2005). Distinctions are thus made between the phenolic acids, flavonoids, stilbenes, and lignans. The flavonoids, which share a common structure consisting of 2 aromatic rings (A and B) bound together by 3 carbon atoms forming an oxygenated heterocycle (ring C), may themselves be divided into 6 subclasses as a function of the type of heterocycle involved: flavonols, flavones, isoflavones, flavanones, anthocyanidins, and flavanols (catechins and proanthocyanidins). In addition to this diversity, polyphenols may be associated with various carbohydrates and organic acids as well as with one another (Manach and others 2004; Tapiero and others 2004).

Polyphenols are probably the most investigated molecules of nutritional interest. Expansive prospective studies, as well as small cross-sectional observations and interventions, have scientifically linked this class of molecules to demonstrated beneficial effects in terms of human health. On this basis, numerous studies have tried to evaluate through which mechanisms polyphenols exert their health benefits (Bravo and others 1994; Tapiero and others 2002; Manach and others 2004; Manach and others 2005; Del Rio and others 2009). These molecules have demonstrated several biological effects, as tested *in vitro* or *ex vivo*. They can inhibit the proliferation of cancer cells, reduce vascularization, protect neurons against oxidative stress, and stimulate vasodilation and improve insulin secretion (Ferguson and others 2004; Silva and others 2008).

On the other hand, carotenoids and related compounds are the colors of nature. They consist of a group of over 600 naturally occurring colored pigments that are widespread in plants, but only about 24 commonly occur in human foodstuffs (DellaPenna and Pogson 2006). In the plant, they serve 2 essential functions as accessory pigments in photosynthesis and photoprotection. These roles are achieved through the polyene structure of carotenoids, which allows the molecules to absorb light and to quench, or inactivate, singlet oxygen, and free radicals (Southon and Faulks 2001).

Dietary intake of carotenoid-rich fruits and vegetables has been associated with reduced risk for a variety of common diseases including multiple types of cancer, cardiovascular disease, macular degeneration, and cataract formation (Rock and others 1998). Carotenoids possess antioxidant properties that have been associated with cellular protection (Mayne 2003), regulation of cell growth, differentiation, and apoptosis (Tapiero and others 2004). However, it is now well established that antioxidants undergo substantial metabolism after being ingested by humans in dietary relevant amounts and that concentrations of plasma metabolites after normal dietary intake rarely exceed nanomolar levels (Manach and others 2005).

### Phenolic compounds and carotenoids bioaccessibility and bioavailability

The *in vivo* effects of antioxidants depend not only on their concentrations in fruits and vegetables, but also on their bioaccessibility and bioavailability after ingestion. Many studies have focused on the bioavailability of these compounds after their ingestion (Manach and others 2005; Parada and Aguilera 2007; Pérez and others 2009).

Understanding the concept of *bioavailability* is essential to all persons involved in food production and nutritional assessment as well as for determining diet and health relationships (Manach and others 2004; Manach and others 2005). Bioavailability is defined as the proportion of an antioxidant that is digested, absorbed, and utilized in normal metabolism; however, measurement of bioavailability relies heavily upon estimates of amounts of antioxidant absorbed. On the other hand, *bioaccessibility* is a commonly used term defined as the amount of an ingested nutrient that is available for absorption in the gut after digestion (Hedren and others 2002). In these terms, the bioavailability strictly depends on the bioaccessibility.

In general, the absorption and transport processes of many of the potentially bioactive components of fruits and vegetables are complex and not fully understood; thus, prediction of their bioavailability is problematic. In this review, 2 major groups of antioxidants are considered: the phenolic compounds and carotenoids.

**Figure 1:**
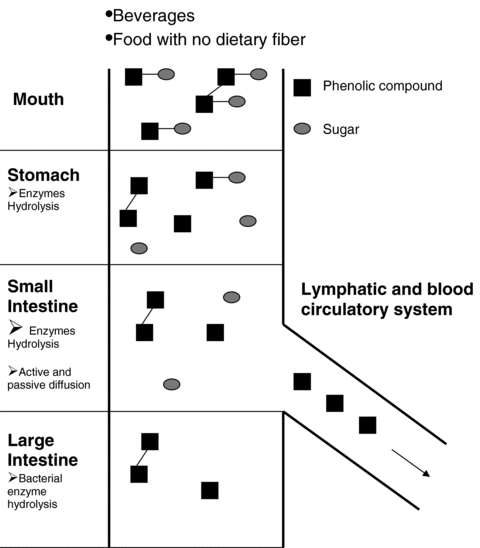
General human bioabsorption of phenolic compounds contained in beverages or similar foods poor in fiber.

Initially, the absorption of polyphenols from the diet was believed to be negligible, given that the majority of food flavonoids are bound to glycosides (Robles-Sardin and others 2010). It was expected that only the aglycones could pass freely into the blood stream from the gut wall, because no specific enzymes are secreted in the gut that can cleave glycosidic bonds (Manach and others 2005). Recent studies, however, have demonstrated that the bioavailability of specific flavonoids and phenolic compounds is much higher than previously believed. The overall process is shown in Figure 1.

The metabolism of phenolic compounds from beverages and food lacking dietary fiber practically starts in the lumen of the small intestine and postabsorption modifications also occur in the liver and other organs (Manach and others 2005; Mullen and others 2006). In the case of polyphenols, as in the flavonoids, the majorities of these compounds are absorbed in some form from the intestine after consumption from food and beverages and can pass through the gut wall into the blood stream (Lafay and Gil-Izquierdo 2008). In order for the absorption of flavonoids from the small intestine to occur, the sugars (glycosides) that are attached to the flavonoid skeleton must first be removed (Pérez and others 2009). This process is controlled by the action of enzymes manufactured in the small intestine (for example, mammalian β-glucosidase), resulting in the release of the flavonoid skeleton (the aglycone) from its sugar (Denny and Buttriss 2007). Furthermore, there is also a growing appreciation for the fact that a significant portion of ingested dietary flavonoids and related compounds is not absorbed in the small intestine but rather passes to the large intestine. Here, they are degraded by the colonic microflora to simple phenolic acids, which can be absorbed into the circulatory system or exert antioxidant activity in the intestine environment (Del Rio and others 2009).

Scientists have found that the type of sugar attached to the flavonoid skeleton determines the site and the extent of absorption of glycosylated flavonoids (Donovan and others 2006). The location at which the sugar is attached to the flavonoid skeleton affects the mechanism by which glycosylated flavonoids are absorbed (Donovan and others 2006). Once the sugars have been removed from the skeleton for absorption, flavonoids are further metabolized in the gut and subsequently in the liver and kidneys to produce a vast number of modified molecules, known as flavonoid secondary metabolites. This process involves further conjugation of the flavonoid through the joining of glucuronate, sulfate, or methyl groups. In the intestine, this process is controlled by enzymes produced by the gut bacteria that play an important role in the metabolism of plant bioactive compounds (Denny and Buttriss 2007).

Flavonoid secondary metabolites can be detected in the blood and urine following the consumption of flavonoid-containing fruits and beverages, but only very small quantities of nonconjugated flavonoids in their original form can found in the same specimens (Kroon and others 2004). This implies that flavonoid secondary metabolites enter the circulation, and evidence suggests that it is these secondary metabolites, rather than the native flavonoids found in fruits and vegetables, that exert biological effects in the body (Kroon and others 2004; Denny and Buttriss 2007).

Research concentrated on the polyphenolic compounds found in fruits, vegetables, and beverages indicates that these molecules are not those that are transported through the human body in the circulatory system, or reach body tissues to elicit bioactive effects (Donovan and others 2006). Instead, it is their metabolites, formed in the small intestine and hepatic cells, and low molecular weight catabolic products of the colonic microflora that elicit antioxidant effects. Understanding transport and modification of these compounds certainly carries interest for drug discovery but also for dietary prevention of disease (Del Rio and others 2009).

On the other hand, the phenolic acids, the main phenol compounds in the human diet, are categorized in 2 large classes: benzoic acid derivatives and cinnamic acid derivatives (Konishi and others 2006). Both classes of molecules have been found to be present in fruits and vegetables at varying concentrations (Joshipura and others 2001; Saura-Calixto and others 2007). However, improving our knowledge of phenolic acid bioavailability is an essential step in understanding their health effects. Even if phenolic acids are the main phenol compounds consumed, the bioavailability of these molecules has not received as much attention as that of flavonoids (Lafay and Gil-Izquierdo 2008).

Phenolic acids in the aglycone form are generally absorbed in the upper part of the gastrointestinal tract (Saura-Calixto and others 2007). Recently, it was shown that the stomach constitutes an active absorption site of numerous phenolic acids as gallic caffeic, ferulic, coumaric, and also chlorogenic acids can be absorbed from the stomach (Konishi and others 2006; Lafay and others 2006; Lafay and Gil-Izquierdo 2008a). This fact explains the rapid absorption of these compounds ranging from 1 to 2 h after intake of fruits and vegetables. The small intestine constitutes another absorption site. However, aglycone phenolic acids can be absorbed to different degrees—19.1% compared with 56.1% for caffeic and ferulic acids, respectively. In general, when the phenolic acids are esterified, the bioavailability decreases, reaching only 0.3% to 0.4% absorbed from the original intake. This is because esterified phenolic acids must be hydrolyzed in the enterocytes before reaching the blood circulation and the enzymatic machinery of these intestinal cells is not efficient enough to hydrolyze the ester bonds (Adam and others 2002; Lafay and others 2006).

**Figure 2:**
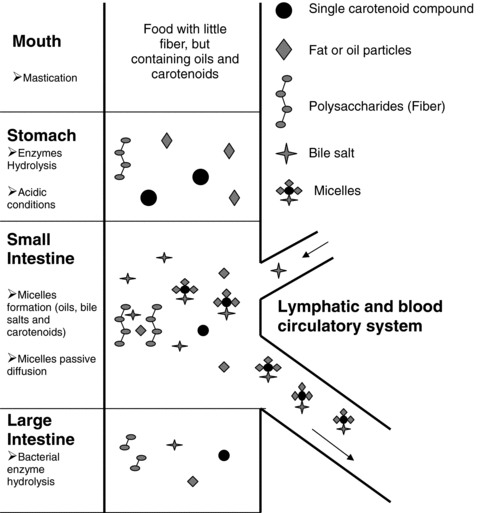
General human bioabsorption of carotenoids contained in food poor in dietary fiber.

Carotenoids are hydrophobic and their absorption depends upon efficient release from the food matrix and subsequent solubilization by bile acids and digestive enzymes, culminating in their incorporation into micelles (Figure 2). Carotenoids are disassociated from their native environment in the plant tissue during food processing and digestion (acidic conditions and enzymatic hydrolysis) in the stomach (Khachik and others 2002). Also, carotenoids must also be released from their native environment during mastication, where mechanical forces and saliva enzymes disrupt tissues and cellular compartments (Parada and Aguilera 2007). The main part of carotenoid metabolism occurs in the small intestine where they must also be dissolved in dietary lipids before they can be absorbed. Dietary lipids have been considered to be an important factor for stimulation of bile flow into the intestine and micelle formation (Furr and Clark 1997; Roodenburg and others 2000). Lipids are capable of attaching to carotenoids due their hydrophobic character, but also may attach to water molecules that, in conjunction with bile salts, result in the formation of micelles and solubilized carotenoids in the system (Hof and others 2000). This allows carotenoids to be absorbed passively from the micellar phase trough the lumen of the intestine to the lymphatic and blood circulatory system. However, it is unknown if all the carotenoids present in a mixed micelle are absorbed, or whether some are left behind in association with unabsorbed bile salts and cholesterol to be absorbed more distally or lost to the large intestine where they can exert their antioxidant properties.

Because it is recognized that the carotenoids are not actively absorbed by the gut but are passively absorbed along with lipids, the efficiency of absorption of carotenoids is dependent on dissolving lipophilic molecules into dietary lipids (Hof and others 2000). This may happen during food preparation and during the digestive processes. It is now recognized that this is a key process in absorption and may well be the single most important factor governing the rate and limit of absorption. It is not surprising, therefore, to find greater bioavailability from heat-treated foods that have also been coprocessed with oils (Stahl and Sies 1992; Gartner and Gellerstedt 1997). However, limited information exists on the bioavailability of dietary lipids and carotenoids entrapped in the food matrix. Soluble dietary fiber in the gut could attenuate the absorption of dietary fats and may, therefore, also inhibit the absorption of carotenoids as lipid soluble compounds (Rock and others 1998; Unlu and others 2005).

### Antioxidant releasing and bioaccessibility

The first physical transformation of fruit and vegetable matrices during eating occurs in the mouth, and mastication is considered the initial step in the digestion of foods. Mastication consists of grinding food into small pieces and impregnating these pieces with saliva to form a bolus that is able to be swallowed. Decreasing the particle size enlarges the surface area available for action by digestive enzymes, thus increasing the overall digestion efficiency and the gastrointestinal absorption of antioxidants (Kulp and others 2003).

As stated before, bioaccessibility is defined as the amount of a food constituent that is present in the gut, as a consequence of the release of this constituent from the solid food matrix and that may be able to pass through the intestinal barrier. Only antioxidants released from the fruit and vegetable matrix by the action of digestive enzymes (small intestine) and bacterial microflora (large intestine) are bioaccessible in the gut and therefore potentially bioavailable (Saura-Calixto and others 2007). Bioaccessibility is not taken into account in studies regarding the bioavailability of polyphenols. Moreover, most studies on polyphenol bioavailability use mainly pure single molecules (isolated from food or chemically synthesized), some beverages, and single foods; however, the bioavailability from whole foods may be substantially different (Manach and others 2005).

### The influence of dietary fiber on the absorption of carotenoids and phenolic compounds

There is ample evidence that the physical state of the food matrix plays a key role in the release, mass transfer, accessibility, and biochemical stability of many food components (Aguilera 2005; Parada and Aguilera 2007). Antioxidants are often located in natural cellular compartments or within assemblies produced during processing. In either case, they need to be released during digestion so that they can be absorbed in the gut (Parada and Aguilera 2007). Furthermore, less is known about the interactions of antioxidants with other food components, such as dietary fiber. It is known that dietary fiber can reduce the bioavailability of macronutrients, especially fat, and some minerals and trace elements in the human diet. Because it was demonstrated in humans that pectin strongly decreased the bioavailability of β-carotene (Rock and Swendseid 1992), dietary fiber is suspected to also affect the absorption of other carotenoids and probably that of α-tocopherol and polyphenols compounds.

In general, the 2 main effects of dietary fiber in the foregut are to prolong gastric emptying time and to retard absorption of nutrients. Both are dependent on the physicochemical form of the fiber, and in particular, on its influence on digesta viscosity.

Dietary fiber can act in the small intestine in 3 main physical forms: as soluble polymer chains in solution, as insoluble macromolecular assemblies, and as swollen, hydrated, sponge-like networks (Eastwood and Morris 1992). The principal physiological effect of dietary fiber in the small intestine is to reduce the rate (and in some cases the extent) of release of nutrients or antioxidants (Brownlee and others 2006). The dominant factors involved in the influence of dietary fiber on antioxidant digestion are (i) physical trapping of antioxidants within structured assemblies such as fruit tissue, and (ii) enhanced viscosity of gastric fluids restricting the peristaltic mixing process that promotes transport of enzymes to their substrates, bile salts to unmicellized fat, and soluble antioxidants to the gut wall (Montagne and others 2003). Secondary factors may include binding of bile salts (and perhaps enzymes) to specific fiber components and inhibition of diffusion across the unstirred layer (Adiotomre and others 1990; Eastwood and Morris 1992).

The rate of release of antioxidants from fibrous particles into the surrounding intestinal fluid is inversely proportional to particle size and is directly proportional to solute gradient (Adiotomre and others 1990; Cummings and others 2004). It is also affected by the following factors: the physical state of the solute (for example, whether it is present in solid form or is already dissolved in water trapped within the particle); the physical structure of the particle (for example, whether it is readily deformed, like a sponge, so that dissolved solids can be squeezed out by peristaltic contractions, or rigid, so that solutes must diffuse out); and the surface properties of the particle (for example, surface-tension effects) (DeVries 2004). The concentration of antioxidants within the continuous aqueous phase is constantly depleted by enteric absorption and replenished, as outlined above, by the release of material from food particles. The progress of these sequential release processes is, of course, also influenced by transit time (that is, the duration of exposure to a particular absorptive surface or digestive environment) (Eastwood and Morris 1992).

In addition to increasing the viscosity of the luminal contents, dietary fiber may reduce rates of antioxidant absorption mainly by physically trapping the antioxidants within the fiber matrix in the chyme. The chyme may be considered a 2-phase system with a discontinuous particulate phase dispersed in a continuous liquid phase. Antioxidants trapped within the particles must first be released into the continuous solution phase before they can be absorbed through the gut wall (Morris 1990; Eastwood and Morris 1992). Chemical interactions between polar groups from polyphenols and fiber polysaccharides may occur, but this has not been well studied (Eastwood and Morris 1992; Pérez and others 2009).

A significant number of studies concerning carotenoid absorption have been published in recent years. Most of these studies discuss the modulating factors previously reported. All of these data demonstrate the significant role exerted on carotenoid bioavailability, for example, by the physical properties of the food matrix that are able to influence digestive processes and thus absorption.

Zhou and others (1996) suggested that the matrix, probably pectin-like fibers, and the crystalline form of carotenoids in carrot chromoplasts were the primary factors that reduced the relative bioavailability of carotenoids from carrots (so-called “incomplete release”). The effect of dietary fiber as pectin on β-carotene response after supplementation is known from earlier work. Studies with chicks and humans suggested that the addition of pectin to chow or test meal reduced the bioavailability of β-carotene (Hoffmann and others 1999). Riedl and others (1999) also showed that pectin decreased the bioavailability of β-carotene by 42%. In addition, Torronen and others (1996) showed a 70% difference in the bioavailability of β-carotene from raw carrots compared with carrot juice consumed by well-nourished adult females for 6 wk, although the difference was not significant. In another study, the relative bioavailability of β-carotene from vegetables compared with purified β-carotene ranged between 3% and 6% for green leafy vegetables, 19% and 34% for carrots, and 22% and 24% for broccoli (Micozzi and others 1992; Castenmiller and others 1999; Hoffmann and others 1999; Porrini and Riso 2008). This has also been widely demonstrated for lycopene (Porrini and others 1998) in tomato products (for example, tomato puree and paste are more bioavailable sources of lycopene than raw tomatoes). The same is true for β-carotene in spinach (Rock and others 1998) in which the plasma response after the ingestion of pureed and thermally processed spinach was observed to be higher than that measured after the intake of raw vegetables. The presence of dietary fiber in vegetables and fruits may explain in part the lower bioavailability of carotenoids from plant foods.

**Figure 3:**
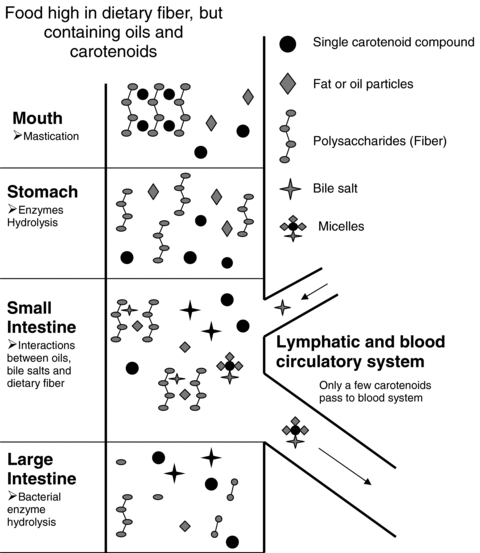
General human bioabsorption of carotenoids contained in foods with high contents of dietary fiber.

A general overview of the interference of dietary fiber in the absorption of carotenoids is shown in Figure 3. The release of carotenoids from plant foods occurs only when the cells in the fruit and vegetables matrix are disrupted, as is usually the case during food preparation, processing, and/or mastication, but not during digestion, at least in the human ileum (Hoffmann and others 1999; Hof and others 2000; van het Hof and others 2000; Edwards and others 2002; Faulks and Southon 2005). The extent of release from the fruit and vegetable matrix is highly variable depending on whether carotenoids are noncovalently bound to protein or fiber, dissolved in oil (as in corn, avocado, or palm oil), or in crystalline form (carrots), making their optimal absorption difficult to achieve (Deming and others 1999; Yeum and others 2002; Zaripheh and Erdman Jr 2002). The role of fiber in fruit matrices where carotenoids are imbibed plays a critical role in adequate absorption (Porrini and Riso 2008). The interactions of carotenoids and specific components of dietary fiber are not clear. The bioaccessibility of carotenoids is interrupted probably because of micelle formation, necessary for the absorption of lipophilic substances, due the disturbance by viscous polysaccharides. It is suggested that fiber interferes with micelle formation by partitioning bile salts and fat in the gel phase of dietary fiber. In other words, the fiber may entrap the lipids and bile salt molecules, thereby avoiding micelle formation with carotenoids, which may block the passive absorption in the small intestine. Furthermore, dietary fiber increases the viscosity of the intestinal content. This results in reduced absorption of antioxidants because of slowed enzymatic activity in the pancreas and increased difficulty in contacting intestinal enterocytes. All the nonabsorbed carotenoids and dietary fiber along with entrapped lipids and bile salts pass to the large intestine, where the polysaccharides are hydrolyzed by bacterial enzymes and the carotenoids may exert their antioxidant activity in the large intestine environment.

Phenolic compounds appear as quantitatively and qualitatively important constituents of indigestible polysaccharides, such as dietary fiber present in fruits and juices (Bravo and others 1994) and other beverages such as beer and wine (Saura-Calixto and Díaz-Rubio 2007; Diaz-Rubio and Saura-Calixto 2009). Phenolic compounds associated with soluble dietary fiber may present different structures, including soluble flavonoids and phenolic acids. The main phenolic compounds associated with dietary fiber in wine are flavan-3-ols and benzoic acids, (Saura-Calixto and others 2007), while in beer are flavonoids, followed by hydroxycinnamic acids linked to arabinoxylans (Diaz-Rubio and Saura-Calixto 2009). Phenolic compounds therefore appear as important constituents of insoluble dietary fiber, which is mainly due to their ability to chemically interact and form complexes with protein and polysaccharides previously generated in the fruit maturation process or the chyme of the gastrointestinal tract (Pérez and others 2009).

In the case of beverages such as wine, phenolic compounds contained in the liquid matrices are promptly bioaccessible and in a condition to exert their beneficial effects on the gastrointestinal tract, but this is not the case for phenolic compounds contained in solid matrices such as those in fruits and vegetables.

**Figure 4:**
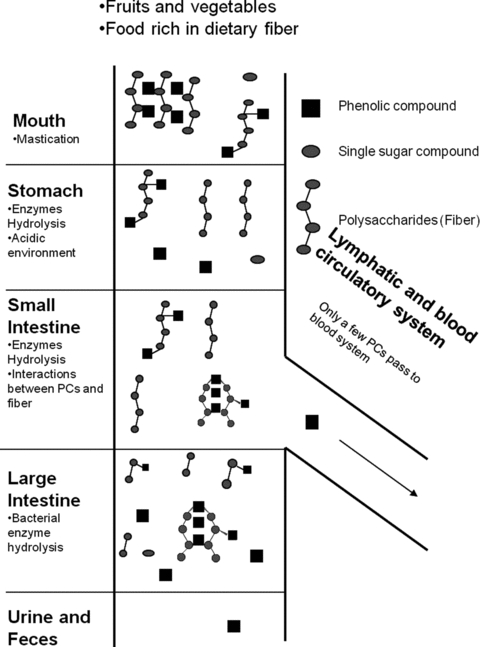
General human bioabsorption of phenolic compounds contained in foods rich in dietary fiber, such as fruits and vegetables.

Phenolic compounds must first be extracted to be bioaccessible and then potentially bioavailable (Tagliazucchi and others 2009), but dietary fiber can interfere with their bioavailability during digestion processes as shown in Figure 4. The gastrointestinal tract may be considered as an extractor where both the mechanical action during mastication in the mouth and the chemical action during the digestive phase in the stomach and intestine contribute to the extraction of phenolic compounds from solid matrices such as fruits and vegetables (Lafay and Gil-Izquierdo 2008). In particular, the mechanical action of mastication mediates the breakdown of fruits cells with the release of the phenolic compounds contained in vacuoles and those linked weakly to the cell wall. The polyphenols linked more closely to the cell wall, especially in the skin cells, are released during the digestive gastro-pancreatic phase as a consequence of the actions of the acidic environment of the stomach and of the alkaline environment of the intestine (Tagliazucchi and others 2009; Del Rio and others 2009). Only a minor portion of low molecular weight phenolic compounds (monomers or oligomers) are able to pass through the gut wall into the small intestine. Unabsorbed low molecular weight phenolic compounds and polyphenols associated with dietary fiber, which account for a major part of dietary polyphenols, are not bioavailable in the human upper intestine and reach the colon, where they become fermentable substrates for bacterial microflora along with indigestible carbohydrates and protein (Manach and others 2005). Phenylacetic, phenylpropionic, and phenylbutyric acids, urolithin A, and urolithin B are absorbable metabolites of polyphenol colonic fermentation that may exert systemic effects (Rechner and others 2004). Nonabsorbable metabolites and nonfermented phenolic compounds remain in the colonic lumen, where they may contribute to a healthy antioxidant environment by scavenging free radicals and counteracting the effects of dietary pro-oxidants (Goñi and Serrano 2005). On the other hand, some polyphenols may be excreted in the feces (Bravo and others 1993).

Polyphenols bound to dietary fiber need to be hydrolyzed by enzymes in the upper area of the intestine; otherwise, these compounds will not be bioavailable for absorption in the human intestine. Considering that dietary fiber acts as an entrapping matrix and restricts the diffusion of the enzymes to their substrates, most of the polyphenols bound to dietary fiber may end up in the large intestine (Pérez and others 2009). From a nutritional point of view, it is apparent that digestible enzymes do not release completely polyphenols associated to dietary fiber; this suggests that this important fraction of polyphenols will not be bioavailable in the gut and only after colonic bacterial fermentation could be absorbed (Saura-Calixto and Díaz-Rubio 2007).

**Figure 5:**
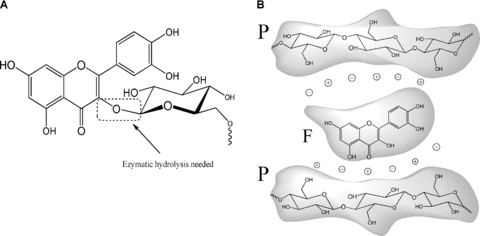
(A) Native flavonoid structure bond to saccharides found in fruits and vegetables. Enzymatic hydrolysis between both molecules during digestion is needed for free absorption of flavonoid in the intestine. (B) Electrostatic and van der Waals interactions between flavonoids (F) and other phenolic compounds with polysaccharide chains (P).

The type of chemical interactions between phenolic compounds and dietary fiber includes the formation of ordered junctions stabilized by arrays of noncovalent bonds between hydroxide groups from phenolic compounds and polar groups from polysaccharide molecules (hydrogen bonds, electrostatic and dipolar interactions, van der Waals attractions) (Eastwood and Morris 1992). The possible interactions and chemical bonds are illustrated in Figure 5. Because these bonds are individually weak, the interactions are stable only above a minimum critical length, and their formation and disruption often occur as sharp, cooperative processes in response to comparatively small changes in, for example, pH or solvent quality in the gastrointestinal tract (that is, the nature and concentration of dissolved solids in the chyme) (Morris 1990). The precisely type of dietary fiber-polyphenols association (electrostatic bonds, hydrogen bonding, van der Waals forces, covalent bonds) remains to be elucidated.

Polyphenols bound to dietary fiber need to be hydrolyzed by enzymes in the upper area of the intestine; otherwise, these compounds will not be bioaccessible for absorption in the human intestine but will be susceptible to degradation by the colonic microflora in the large intestine (Pérez and others 2009). Considering that dietary fiber acts as an entrapping matrix and restricts the diffusion of the enzymes to their substrates, most of the polyphenols bound to dietary fiber may end up in the large intestine.

On the other hand, there is another example of interaction between phenolic compounds and fiber. In bran and aleurone, ferulic acid is mainly bound to arabinoxylans and other cell wall polysaccharides that are able to resist digestion in the upper gastrointestinal tract (Kern and others 2003; Zhao and others 2003). The ferulic acid esters might be hydrolyzed to free ferulic acid *in vivo*. Whether these ferulic acid esters will permeate the mucus layer and reach the esterases in the small intestinal mucosa depends on their molecular size and structure (Mateo-Anson and others 2009). Molecules larger than 30 kDa are not expected to diffuse through the mucus, while diffusion of lower molecular weight molecules is size dependent and influenced by the existence of an electric field (Bravo and others 1994). Zhao and others (2003) showed how differences in the molecular size of ferulic acid sugar esters influences the degree of absorption and the absorption site of ferulic acid within the gut of the rat. When ferulic acid was either esterified to one arabinose or to several arabinoses and xyloses, most of the ferulic acid (60% to 70%) was not absorbed in the small intestine, leading to the conclusion that the major cleavage of ferulic acid esters takes place in the large intestine (Zhao and others 2003). This is in accordance with a previous study (Kroon and others 2004), which reported that the major cleavage of esters bound in hydroxycinnamates such as ferulic acid occurs in the colon by bacterial enzymes.

In summary, the main reasons why phenolic compounds and carotenoids are not bioaccessible due the presence of dietary fiber include the following: (i) they are not well released from fruit and vegetable matrices, (ii) dietary fiber entraps the phenolic compounds during digestion in the upper intestine, and (iii) some antioxidants may be bound to polysaccharides and therefore require enzymatic hydrolysis to be absorbed, which is restricted by the action of dietary fiber matrices formed in the chyme. Finally, all nonabsorbable antioxidants reach the large intestine and remain in the colonic lumen where they may contribute to a healthy antioxidant environment.

## Conclusion

The limited bioavailability of antioxidants present in food from fruit and vegetable matrices is determined by their low bioaccessibility in the small intestine due to the physical and chemical interactions of the antioxidants with the indigestible polysaccharides of cell walls. Even if released during processing and digestion, antioxidants may interact with other food components in the gut by binding to macromolecules such as fiber and forming chemical complexes and colloidal structures that reduce or improve their bioavailability, a subject that needs urgent research. This has remarkable consequences in assessing the nutritional role and real impact of many fruit and vegetable phytochemicals in the prevention and therapy of some chronic human diseases. It is necessary to take serious steps to understand at a higher level the types of interactions and the real repercussions of dietary fiber in the bioabsorption of carotenoids and phenolic compounds in the gastrointestinal tract.

### Future research

The bioavailability of nutrients and bioactive compounds present in fruits and vegetables is presently an extremely important area of food and nutrition research. However, future research is needed to improve the real contribution of fruits consumption to the well-being of consumers. This may be using different process to improve the releasing of antioxidants, especially polyphenols, from solid matrices. Bioavailability i*n vitro* and *in vivo* studies are needed from different fruit matrix with different fiber composition. The role of fiber as a control-released system of bioactive compounds must be studies deeper. On the other hand, considering the fact that dietary fiber interferes with the adequate absorption of antioxidants, an efficient and economic way to improve the antioxidant bioavailability may be to study of new human diet regimens to ensure the right assimilation of antioxidants from meals. These studies could establish times between meals when the absorption of antioxidants is more effective from beverages rather than from solid food. Also establishing times of fiber flow through the digestive system, to estimate when is more adequate to ingest a rich source of antioxidants such as beverages or antioxidants supplements. This simple information would be of great relevance for consumers.
